# Evaluation of medical devices in thoracic radiograms in intensive care unit - time to pay attention!

**DOI:** 10.5935/0103-507X.20160056

**Published:** 2016

**Authors:** Ana Sofia Linhares Moreira, Maria da Graça Alves Afonso, Mónica Ribeiro dos Santos Alves Dinis, Maria Cristina Granja Teixeira dos Santos

**Affiliations:** 1 Department of Radiology, Centro Hospitalar do Algarve - Faro, Portugal.; 2 Department of Emergency and Intensive Care, Centro Hospitalar do Algarve - Faro, Portugal.; 3 Department of Biomedical Sciences and Medicine, Universidade do Algarve - Faro, Portugal.

**Keywords:** Radiography, thoracic, Central venous catheters/utilization, Intubation, intratracheal/instrumentation, Equipment and supplies, Intensive care units

## Abstract

**Objective:**

To identify and evaluate the correct positioning of the most commonly used medical devices as visualized in thoracic radiograms of patients in the intensive care unit of our center.

**Methods:**

A literature search was conducted for the criteria used to evaluate the correct positioning of medical devices on thoracic radiograms. All the thoracic radiograms performed in the intensive care unit of our center over an 18-month period were analyzed. All admissions in which at least one thoracic radiogram was performed in the intensive care unit and in which at least one medical device was identifiable in the thoracic radiogram were included. One radiogram per admission was selected for analysis. The radiograms were evaluated by an independent observer.

**Results:**

Out of the 2,312 thoracic radiograms analyzed, 568 were included in this study. Several medical devices were identified, including monitoring leads, endotracheal and tracheostomy tubes, central venous catheters, pacemakers and prosthetic cardiac valves. Of the central venous catheters that were identified, 33.6% of the subclavian and 23.8% of the jugular were malpositioned. Of the endotracheal tubes, 19.9% were malpositioned, while all the tracheostomy tubes were correctly positioned.

**Conclusion:**

Malpositioning of central venous catheters and endotracheal tubes is frequently identified in radiograms of patients in an intensive care unit. This is relevant because malpositioned devices may be related to adverse events. In future studies, an association between malpositioning and adverse events should be investigated.

## Abstract (pt)

**Objetivo:**

Identificar e avaliar o posicionamento correto dos dispositivos médicos mais comumente utilizados, observados nas radiografias de tórax de pacientes durante a permanência em unidade de terapia intensiva de nosso centro.

**Métodos:**

Foi realizada uma pesquisa bibliográfica quanto aos critérios utilizados para avaliar o posicionamento correto dos dispositivos médicos nas radiografias de tórax. Avaliamos todas as radiografias de tórax realizadas na unidade de terapia intensiva de nosso centro durante um período de 18 meses. Incluíram-se todas as admissões nas quais foi realizada uma radiografia do tórax na unidade de terapia intensiva, nas quais fosse identificável a presença de pelo menos um dispositivo médico. Para análise, selecionou-se uma radiografia por admissão. As radiografias foram avaliadas por um observador independente.

**Resultados:**

De um total de 2.312 radiografias analisadas, 568 foram incluídas neste estudo. Identificaram-se diversos dispositivos médicos, incluindo eletrodos de monitoramento, tubos endotraqueais, cânulas de traqueostomia, cateteres venosos centrais, marca-passos e próteses valvares cardíacas. Dentre os cateteres venosos centrais identificados, 33,6% dos subclávios e 23,8% dos jugulares estavam mal posicionados. Dentre os tubos endotraqueais, 19,9% estavam mal posicionados, enquanto todas as cânulas de traqueostomia tinham posicionamento correto.

**Conclusão:**

Frequentemente se identificam, na radiografia de tórax realizada em pacientes na unidade de terapia intensiva, cateteres venosos e tubos endotraqueais mal posicionados. Isso é importante, pois dispositivos mal posicionados podem se relacionar a eventos adversos. Estudos futuros devem investigar possíveis associações entre o mau posicionamento dos dispositivos e eventos adversos.

## INTRODUCTION

Thoracic radiograms are one of the main auxiliary diagnostic exams performed in intensive care units (ICU).^(1)^ However, according to the American College of Radiology,^(2)^ the daily use of thoracic radiograms is not appropriate, except when there are changes in the patient's medical condition or following the placement of a medical device. Thoracic radiograms should be performed after the placement of medical devices because there are potential complications associated with this practice, and the first element that should be evaluated is the absence or presence and correct positioning of medical devices.^(3)^

Erroneous positioning of medical devices has been shown to be related to certain adverse events.^(2,3)^ Possible adverse events related to the incorrect placement of medical devices may be diagnosed on thoracic radiograms and can include pneumothorax or hemothorax caused by central venous catheters (CVC),^(4,5)^ more commonly by subclavian catheters,^(2)^ cardiac arrhythmias or myocardial rupture that could be caused by low placement of CVC with the tip in the right atrium,^(4,5)^ lung or lobar atelectasis caused by a low placement of an endotracheal tube with bronchial intubation, and an increased risk of extubation when the endotracheal tube tip is placed too high.^(4,6)^

There are several technical limitations in the performance of radiograms in ICU, namely, lack of cooperation from patients in terms of their positioning, making it only possible the execution of antero-posterior incidences in the majority of patients.^(3)^

The aim of this study was to evaluate the types of medical devices commonly used in patients in intensive care units that can be identified on thoracic radiograms and to evaluate their correct positioning, when feasible.

## METHODS

We conducted a literature search for criteria used to evaluate the correct positioning of medical devices on thoracic radiograms, the results of which are summarized in table 1^(4-11)^ and were used for the analysis of the positioning of the devices identified in our study. This study was approved by the Ethics Committee of the Centro Hospitalar do Algarve (CHA), under nº 2264/2015, and there was no need for consent signature.

**Table 1:** Criteria for the correct positioning of medical devices as visualized via thoracic radiograms, based on the literature search. These criteria were applied to assess the accuracy of the positioning of devices in the present study

Endotracheal tube	5cm above the carina or immediately above the aortic arch, with the head in a neutral position If the carina is not visible, the distal end of the tube should be at the level of the medial borders of the claviculae
Tracheostomy tube	Equidistant between the stoma and the carina (generally D3)
Nasogastric and feeding tubes	Cross the mediastinum, in intra-esophageal topography The nasogastric tube passes the cardia and the side holes should be at the level of the gastric antrum The feeding tubes should have their distal end in the second portion of the duodenum
Central venous catheters	Ideally positioned in the superior vena cava Inferior to the junction of the brachiocephalic veins, at the level of the first intercostal space Superior to the cavo-atrial junction, at the level of the inferior border of the intermediate bronchia
Prosthetic cardiac valves	Requires at least 2 incidences to evaluate
Pacemakers Implantable cardioverter defibrillators	The accurate evaluation of the lead position requires at least 2 incidences
Thoracostomy tubes (drains)	Tube should be at the surface of the expanded lung, between the 2 pleuras (for correct visualization 2 incidences are needed) Fourth intercostal space, at the level of the anterior or medium axillary line Side hole should always be medial to the internal margin of the ribs Antero-superior orientation - for pneumothoraxes Postero-inferior orientation - for liquid collections

We reviewed all thoracic radiograms that were performed in the ICU of the Faro Pole of CHA over a period of 18 months (01/01/2014 to 30/06/2015), and all the patients (admissions) who had a thoracic radiogram during their stay in the ICU were included in this study (Table 2). The evaluation was performed by ICU admission and not by patient, as a small number of patients had more than one admission over the 18-month period studied.

**Table 2:** Inclusion and exclusion criteria used to select the radiograms for analysis of the medical device placement

Inclusion criteria	Admission with at least one thoracic radiogram performed in the intensive care unit One radiogram was selected for each patient admission When multiple radiograms were performed per admission, the first radiogram that had the highest number of medical devices was chosen
Exclusion criteria	Absence of identifiable medical devices on the thoracic radiogram

One radiogram per patient was selected for analysis because some patients had multiple thoracic radiograms during their stay in the ICU. The selection criteria used was the first radiogram performed that presented the highest number of identifiable medical devices to avoid any potential selection bias toward selecting radiograms with malpositioned versus correctly positioned devices.

The absence of identifiable medical devices on the radiogram was an exclusion criterion (Table 2).

Selection and analysis of the radiograms was performed by one independent observer from the ICU. When doubt arouse about the classification of the placement of devices, the radiogram in question was evaluated by three of the authors to counteract any potential biases.

We analyzed the type of devices that were more frequently identified and, when possible, the positioning of these devices. When a suboptimal radiogram was obtained for evaluating the medical devices, in particular for endotracheal tubes, a 2cm range was used to account for the patient's head position.

## RESULTS

During the 18 months of the study, there were 755 patients admitted to the ICU (313 females and 442 males), and 2,312 thoracic radiograms were performed on 572 of the admitted patients. All 2,312 radiograms were reviewed to select the first radiogram from each patient (572 radiograms) that had the highest number of identifiable medical devices. After applying the exclusion criteria to the selected radiograms, 4 were excluded: 3 because of an absence of identifiable medical devices and 1 due to the patient's severe scoliosis, which prevented the accurate evaluation of the medical device placements.

A total of 568 radiograms were analyzed for the presence of medical devices, and their positioning was evaluated when possible.

The multitude of medical devices identified on the radiograms is reported in table 3 along with the relative frequency of each device.

**Table 3:** Type of devices identified and their relative frequency

Devices	%
Central venous catheters	84.5
Monitoring leads	83.3
Endotracheal tube	73.6
Gastric tube	57.6
External ventilation support	50
Drains	14.3
Esternotomy material	4.2
Surgical clips	3.5
Tracheostomy tube	3
Pacemaker	1.9
Multifunction paddles	0.9
Osteosynthesis material	0.9
Prosthetic cardiac valves	0.9
Temporary pacemaker	0.5
Implantable cardio-defibrillators	0.2
Thermometer	0.2

In the current study, all of the radiograms were acquired with an antero-posterior incidence. Therefore, the evaluation of the correct positioning of some of the identified devices was not feasible, namely, for pacemakers. Consequently, we were only able to analyze the accuracy of the positioning of CVC, endotracheal tubes and tracheostomy tubes.

**Figure 1:**
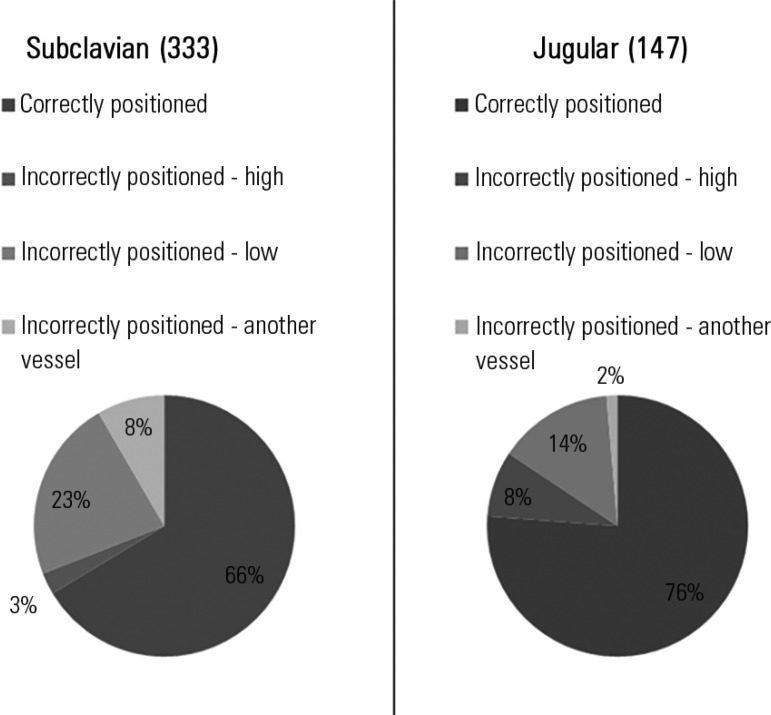
Distribution of the percentages of the types of positions of central venous catheters.

We identified 480 CVC, 333 of which were subclavian and 147 were placed in the jugular. Of the subclavian CVC, 33.6% were malpositioned, as were 23.8% of the jugular CVC (Figure 1).

**Figure 2:**
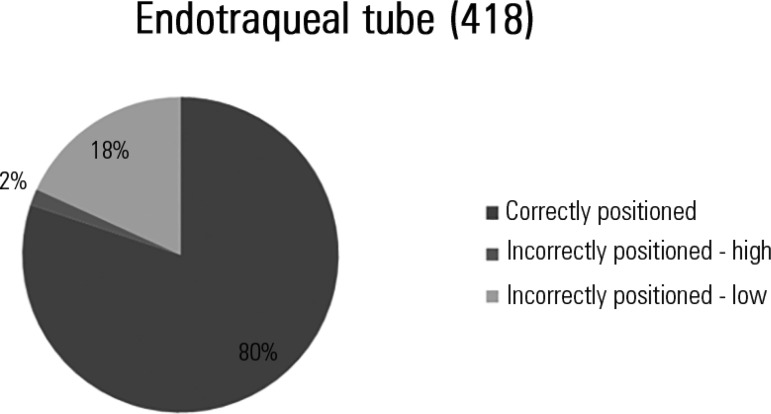
Distribution of the percentages of the types of positions of endotracheal tubes.

We identified 418 endotracheal tubes, 19.9% of which were malpositioned; all of the identified tracheostomy tubes were correctly placed (Figure 2).

## DISCUSSION

A thoracic radiogram is an essential tool for the evaluation of medical devices immediately after placement, especially in patients in the ICU. A physician should request a thoracic radiogram when the expected findings, either positive or negative, could alter the approach to treating the patient.^(12-18)^ In this context, a positive finding would be that a device is malpositioned, which would merit repositioning the device to prevent the development of adverse events, while a negative finding would be that a device is positioned correctly.

This is the first study to analyze medical devices in the ICU of our institution, and its strengths are that it had a substantial sample size: 2,312 thoracic radiograms were reviewed, and 568 of these were analyzed. Furthermore, the evaluations were performed by professionals from outside of the ICU, which decreased the risk of potential bias.

The study also had some limitations. For example, the study was retrospective. It also involved imperfect technical conditions that are inherent to exams performed on patients in the ICU, such as the antero-posterior incidence, the lack of cooperation from and inappropriate positioning of the patient, and the impossibility of achieving a neutral head position.

Due to the aforementioned technical difficulties, all radiograms were performed using an antero-posterior incidence, which limited the ability to investigate the medical devices. For instance, some of the identified devices require at least two incidences to accurately determine their location, namely, prosthetic cardiac valves, pacemakers, implantable cardioverter defibrillators and thoracic drains. In addition, there is a need for complementary radiograms of the abdomen to determine the position of gastric tubes, which are denominated as such because it was not possible to accurately assess whether the distal extremity of these tubes was in the stomach or in the duodenum, nor was it possible to assess whether the proximal extremity was in the mouth or in the nose.

Due to these limitations, it was only possible to analyze the correct positioning of CVC, endotracheal tubes and tracheostomy tubes.

We found a relatively high frequency of malpositioning, with incorrectly placed CVC found in 30.6% of cases (with some series recording from 10% up to 40% misplaced CVC)^(2,5,6)^ and with incorrectly placed endotracheal tubes identified in 20% of analyzed cases (some records report values from 15% up to 28 - 46%).^(2,5,6)^

The association between these findings and possible related adverse events was not evaluated in the present study and should be investigated in future studies. In addition, no risk factors for the malpositioning of devices were sought or evaluated, but this should also be examined in the future.

## CONCLUSION

In an intensive care unit context in our study, only central venous catheters, endotracheal tubes and tracheostomy tubes could be evaluated due to the limitations stated above.

An increased awareness of the use of radiograms as a method to identify and diagnose malpositioned medical devices could prevent the development of adverse events. Thus, the knowledge of correct and incorrect positions of medical devices as visualized via radiograms is essential for making diagnoses of correct medical device positioning, which is crucial because of the relatively high prevalence of incorrectly positioned devices in an intensive care unit population (30% of central venous catheters and 20% of endotracheal tubes were malpositioned in this sample).

In future studies, an association between malpositioning and adverse events should be investigated.
